# Endoscopic Management of Concomitant Malignant Biliary and Gastric Outlet Obstruction

**DOI:** 10.7759/cureus.75635

**Published:** 2024-12-13

**Authors:** Intekhab Hossain, Hannah Jardine, Keeran Bonia, Bradley Evans

**Affiliations:** 1 Surgery, University of Toronto, Toronto, CAN; 2 Surgery, Memorial University of Newfoundland, St. John's, CAN

**Keywords:** advance endoscopy, case report, malignant biliary obstruction, malignant gastric outlet obstruction, self-expandable metal stent

## Abstract

Concurrent malignant biliary and gastric outlet obstruction requires urgent palliative intervention to improve patient quality of life and permit systemic therapy. Traditional management has been surgical gastrojejunostomy and hepaticojejunostomy, two morbid procedures. Comparatively, endoscopic stenting can relieve both sites of obstruction with less complications and quicker recovery. In patients with previous plastic biliary stents in situ, it is crucial for subsequent bilioduodenal obstructions to be managed with proper sequencing and precise stent placement to achieve successful bilioduodenal patency.

We report a case of a 53-year-old male patient who presented with simultaneous jaundice secondary to blocked biliary stent and vomiting due to gastric outlet obstruction at the first part of the duodenum on background of unresectable pancreatic adenocarcinoma. Fourteen months prior, he had a plastic endobiliary stent placed for biliary obstruction secondary to choledocholithiasis, but intraprocedural cholangiogram also revealed a distal common bile stricture with subsequent investigations revealing unresectable pancreatic adenocarcinoma for which he underwent palliative chemotherapy. Duodenal stricture dilation with subsequent duodenal self-expanding metal stent was placed under direct endoscopic vision precisely proximal to the blocked biliary stent. After 48 hours, endoscopic retrograde cholangiopancreatography was then performed through the duodenal stent to exchange the blocked plastic biliary stent for a metal biliary stent. The patient had prompt relief of jaundice and tolerated oral intake by date of discharge post-procedure day two and was initiated on chemotherapy on post-procedure day 12. Endoscopic stenting of concomitant biliary and gastric outlet obstruction can be successful in patients with occluded indwelling plastic biliary stents.

## Introduction

Concurrent biliary and gastric outlet obstruction can present in the setting of advanced periampullary cancer. They are often diagnosed at an advanced stage, narrowing curative treatment options. Advances in endoscopic therapy have allowed for less morbid methods of alleviating both sites of obstruction compared to traditional surgical management with gastrojejunostomy and hepaticojejunostomy. Bilioduodenal strictures are categorized using the Mutignani classification, in relation to the site of duodenal obstruction being proximal to (type 1), at the level of (type 2), or distal to (type 3) the major papilla [1]. Treatment of bilioduodenal obstructions is necessary to improve the quality of life as well as to facilitate chemotherapy administration, for this typically palliative patient population. In this case report, we demonstrate successful endoscopic management of type 1 bilioduodenal stricture in a patient with unresectable pancreatic head adenocarcinoma and occluded plastic biliary stent, with duodenal stricture dilation and duodenal stent placement with subsequent through-duodenal-stent exchange of the biliary stent. We also summarize, from the literature review, treatment methods and considerations of all three types of Mutignani bilioduodenal strictures. This article was previously presented as a meeting abstract at the 2024 Canadian Surgery Forum Meeting on September 28, 2024.

## Case presentation

**Figure 1:**
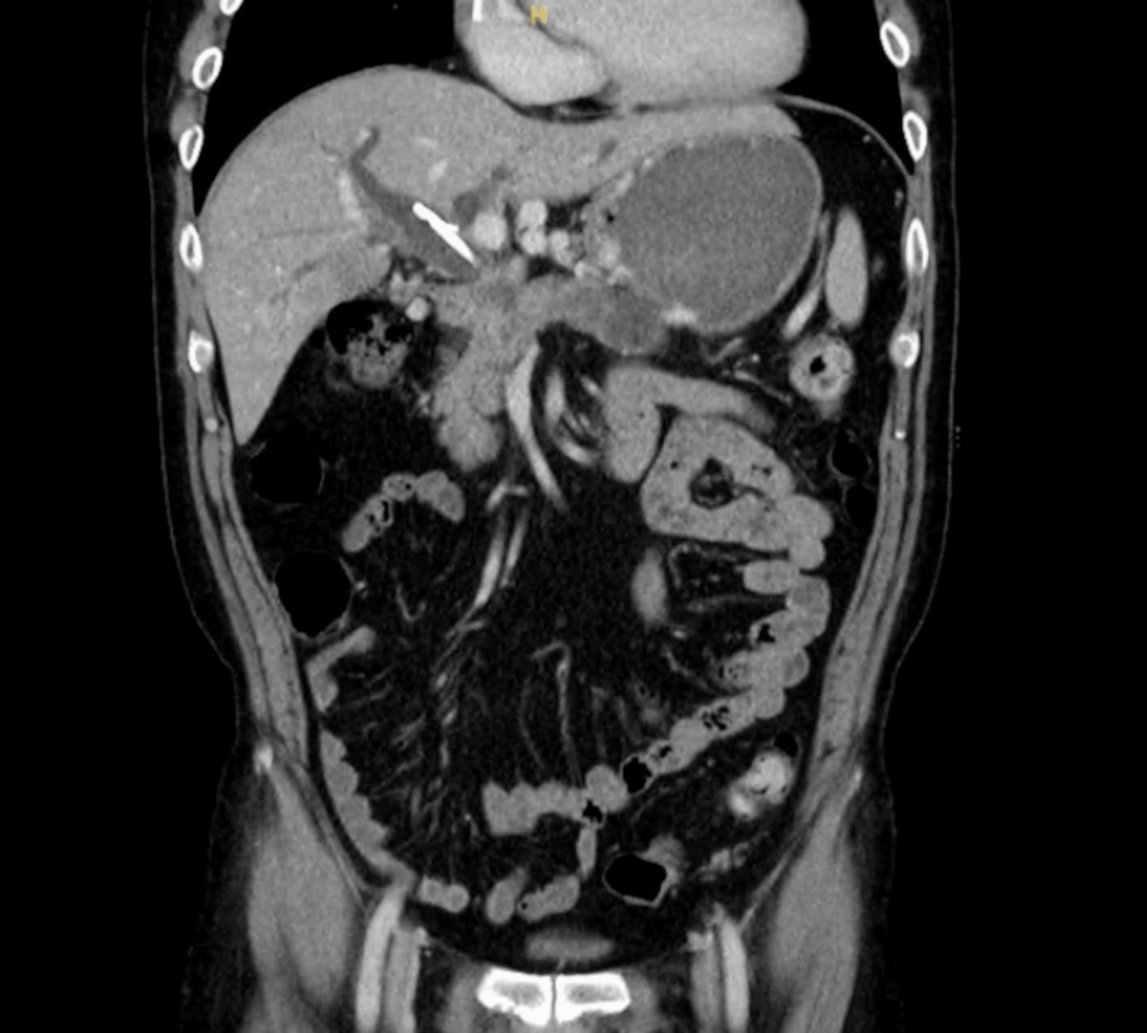
Abdominopelvic CT image of the patient’s type 1 Mutignani stricture with concurrent biliary obstruction and duodenal obstruction proximal to the ampulla CT: computed tomography

**Figure 2:**
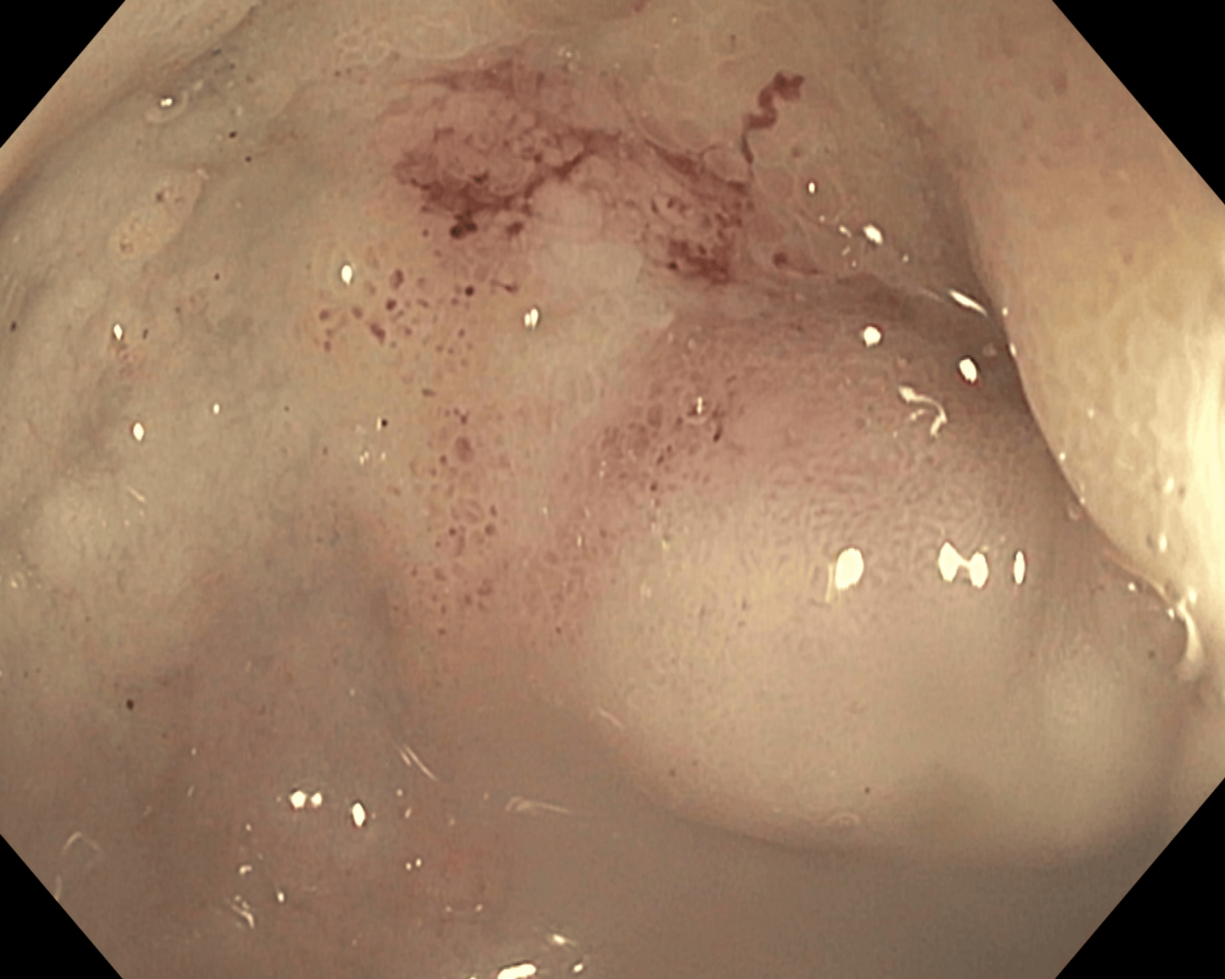
Esophagogastroduodenoscopy view of obstruction at the duodenal cap

**Figure 3:**
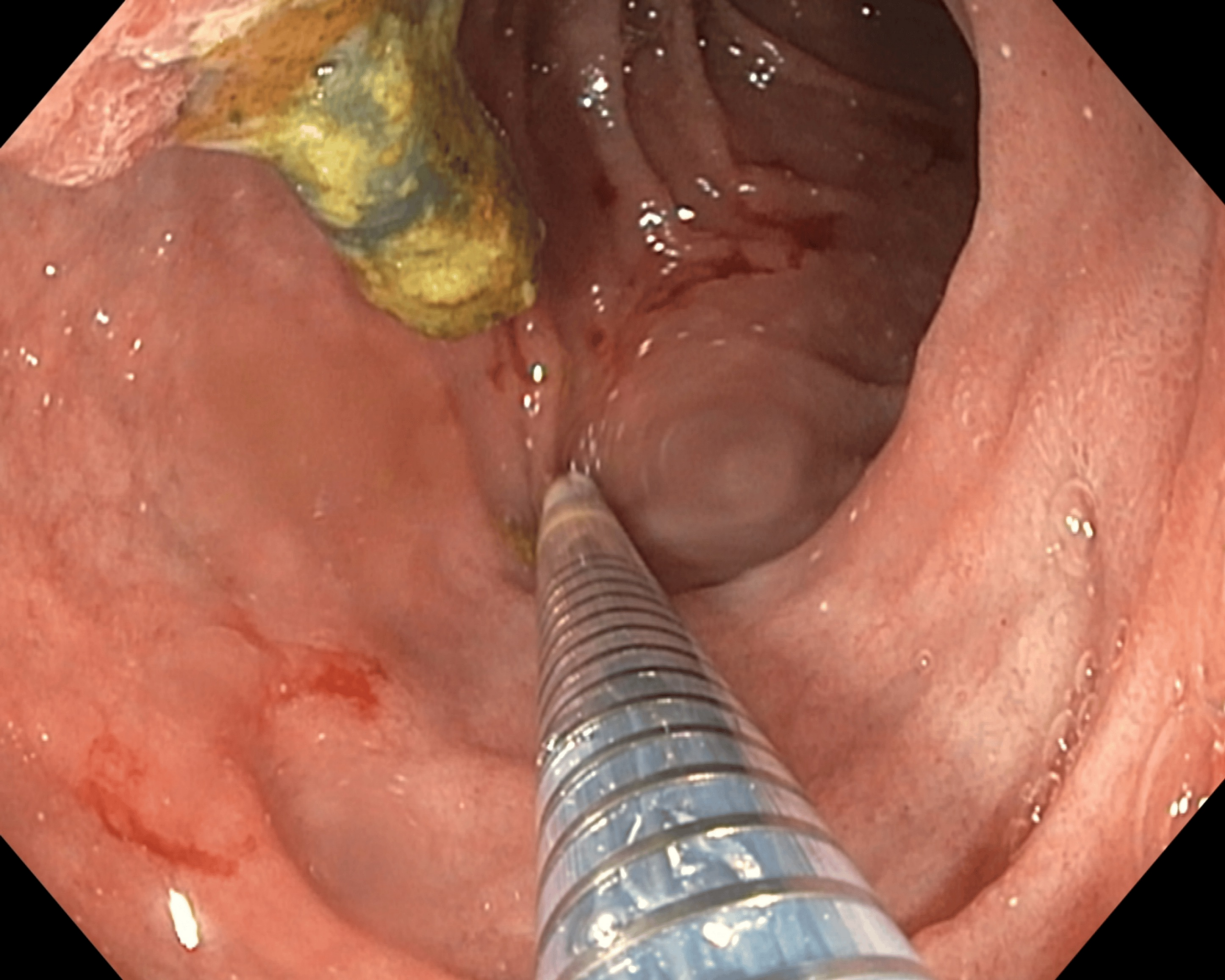
Gastroduodenal stent deployment with occluded common bile duct plastic stent in situ

**Figure 4:**
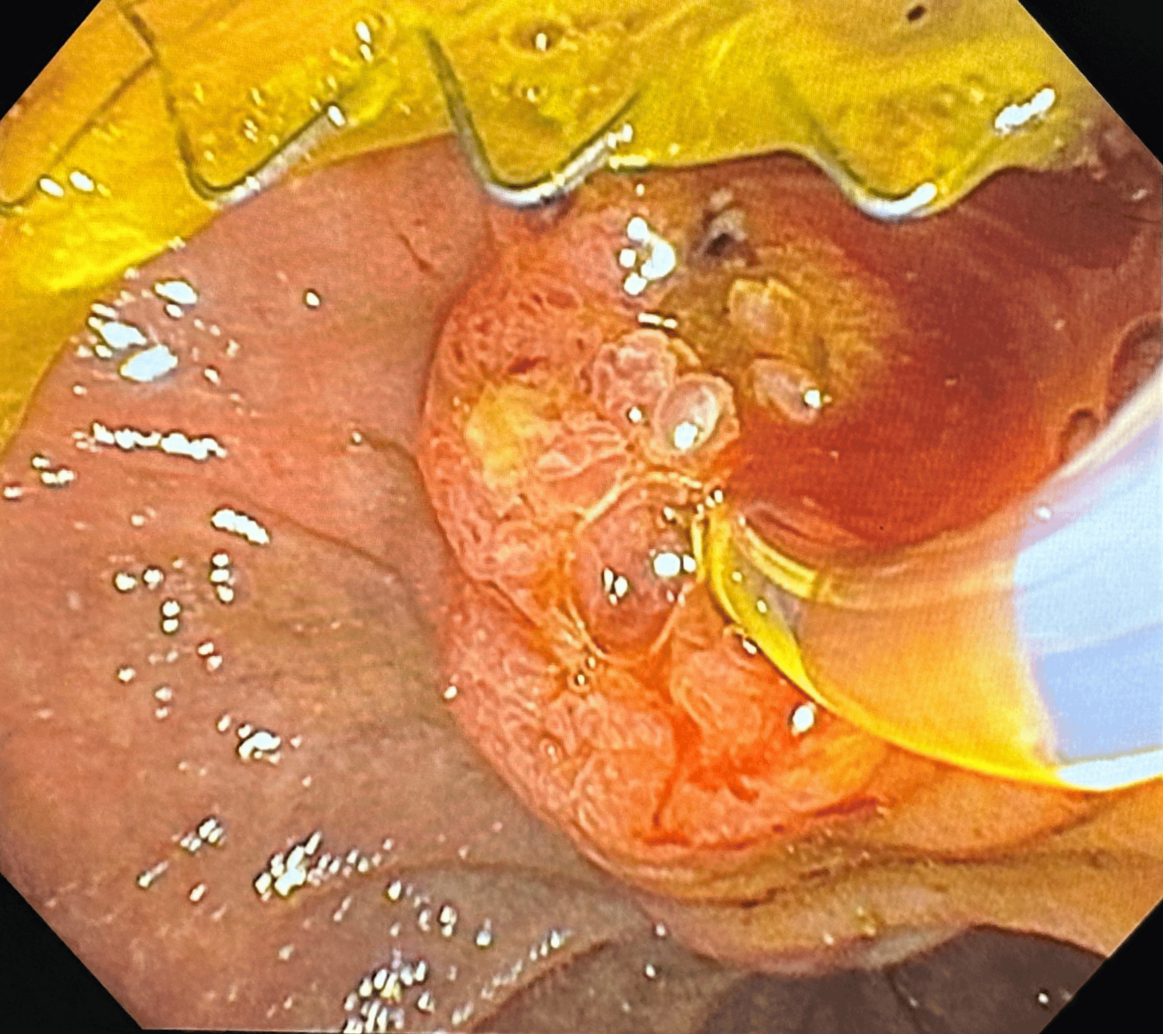
Biliary stent deployment with side viewing duodenoscope through gastroduodenal stent

**Figure 5:**
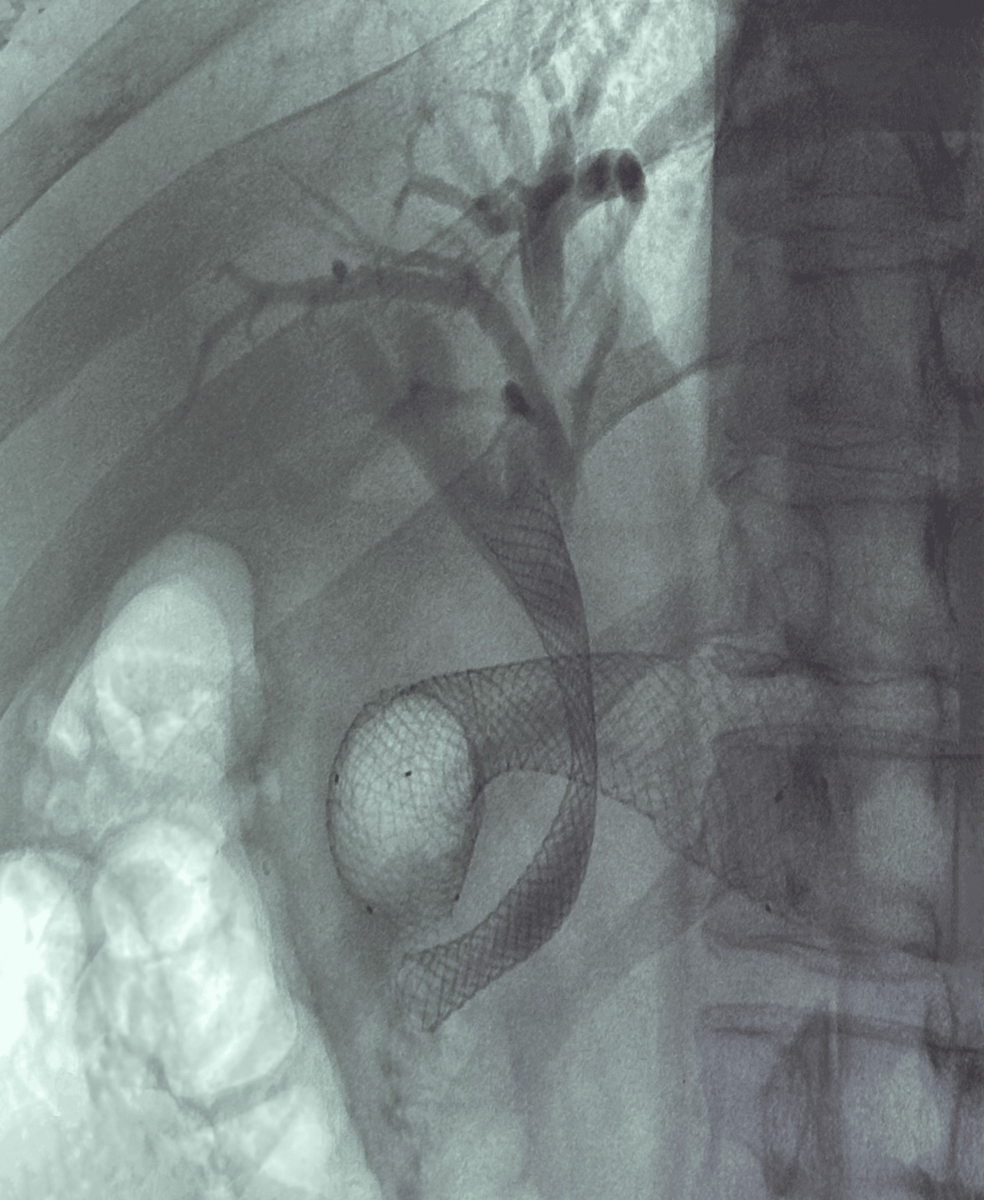
Fluoroscopic image of the gastroduodenal and biliary metal stents in situ during ERCP ERCP: endoscopic retrograde cholangiopancreatography

A 53-year-old male patient presented to the hospital with five days of right upper quadrant abdominal pain and jaundice. Bloodwork demonstrated obstructive jaundice with total bilirubin 178 µmol/L, aspartate aminotransferase (AST) of 220 U/L, alanine aminotransferase (ALT) of 532 U/L, alkaline phosphatase (ALP) of 668 U/L, amylase of 68 U/L, hemoglobin of 144 g/L, white blood cell count of 3.9 x 109/L, platelet count of 167 x 109/L, international normalized ratio (INR) of 1.01, and normal electrolytes and creatinine. Abdominal ultrasound showed biliary obstruction with a dilated 14 mm common bile duct and choledocholithiasis. There were no signs of cholangitis. Given the findings of common bile duct stones on ultrasound imaging and clinical symptoms in keeping with choledocholithiasis, endoscopic retrograde cholangiopancreatography (ERCP) was performed. ERCP revealed several gallstones and a distal common bile duct stricture. Stones were extracted with balloon sweeps, and the stricture was brush biopsied. A 10 French 9 cm plastic endobiliary stent was successfully placed with subsequent normalization in total bilirubin levels for three days. Over the next five days during hospital admission, abdominal and pelvic computed tomography (CT), magnetic resonance cholangiopancreatography (MRCP), and endoscopic ultrasound (EUS) pancreatic biopsy revealed stage IV, unresectable, and metastatic pancreatic head adenocarcinoma with tumor encasing the celiac trunk and hepatic arteries with resultant obstruction of the portal, superior mesenteric, and splenic veins along with a hepatic metastatic deposit. Palliative FOLFIRINOX chemotherapy was initiated and continued for 21 completed cycles until 14 months later, when the patient represented to the hospital with jaundice and vomiting. Bloodwork during this presentation showed recurrent obstructive jaundice with total bilirubin of 74 µmol/L, AST of 178 U/L, ALT of 307 U/L, ALP of 644 U/L, hemoglobin of 135 g/L, white blood cell count of 17.0 x 109/L, platelet count 214 x 109/L, and normal electrolytes, creatinine, and amylase. He was started on intravenous (IV) ceftriaxone and metronidazole for possible cholangitis but remained stable. Abdominopelvic CT and gastroscopy revealed a Mutignani type 1 bilioduodenal stricture, with occluded biliary stent and obstruction at the first portion of the duodenum, in keeping with progression of previously known metastatic pancreatic adenocarcinoma (Figure 1). ERCP was attempted, but the duodenal stenosis could not be traversed (Figure 2). Duodenal stenting was considered, but this presented a unique scenario as there was a high risk of caging in the plastic biliary stent. We managed this case of concomitant malignant biliary and gastric outlet obstruction using through-the-scope (TTS) balloon dilatation to 12 mm using TTS balloon dilation and subsequent placement of a 22 x 90 mm uncovered duodenal self-expanding metal stent (Cook Medical; Bloomington, Indiana, USA) under direct endoscopic view with a therapeutic gastroscope. This allowed duodenal stent placement precisely proximal to the plastic biliary stent (Figure 3). Duodenal stent was left for 48 hours allowing expansion, to facilitate ERCP and prevent stent migration. Successful biliary stent exchange with ERCP through-the-duodenal-stent under fluoroscopic guidance was then performed and a 10 x 60 mm uncovered self-expanding metal biliary stent (Boston Scientific; Marlborough, Massachusetts, USA) was placed (Figures 4-5).The patient tolerated oral intake with relief and normalization of total bilirubin to 26 µmol/L by post-procedure day two and was discharged home post-procedure day three with total bilirubin of 20 µmol/L. Palliative chemotherapy with gemcitabine and nab-paclitaxel was initiated post-procedure day 12. He received four cycles of this chemotherapy regimen and, then due to disease progression, switched to FOLFOX for three cycles. No further interventions were required for the duodenal and biliary stents until his passing five months later due to continued disease progression leading to kidney and liver failure without signs of repeat gastric outlet or biliary obstruction and change of goals of care to comfort measures. 

## Discussion

Patients with unresectable periampullary cancer, cholangitis, or in need of nutritional rehabilitation, presenting with simultaneous biliary and gastric outlet obstruction may be considered for referral to advanced endoscopy services for possible palliative dual gastroduodenal and biliary stent placement, if available. Endoscopic dual gastroduodenal and biliary stent placement is a lower morbidity solution with higher clinical success, shorter time to oral intake, lower procedural costs, and decreased hospital stay compared to surgical gastrojejunostomy and hepaticojejunostomy [2,3] for palliative patients with concomitant malignant biliary and gastric outlet obstruction. This also facilitates early return to systemic therapy.

Endoscopic management technique of bilioduodenal strictures is safe and effective [2] but dependent on the location of the enteric stricture in relation to the ampulla; success rates vary at 94%-100%, 80%-86%, and 100% for Mutignani types 1-3 strictures, respectively [1,3,4]. For type 1 strictures, balloon dilation of the enteric stricture is typically required to allow passage of an ERCP duodenoscope. Subsequently, enteric and, then, biliary self-expanding metal stenting can be performed [3]. Our case posed a unique challenge as the patient presented with an indwelling occluded plastic biliary stent with duodenal stricture, as opposed to de novo biliary obstruction. Thus, it was imperative to place the duodenal stent precisely proximal to the ampulla to avoid caging in the plastic stent and allow biliary stent exchange with a through-the-duodenal-stent ERCP. For type 2 strictures, the most difficult type, biliary stenting with endoscopic or percutaneous transhepatic self-expanding metal stent is preferred prior to enteric stenting [5]. However, if the ampulla cannot be reached, a duodenal metal stent may be placed first, and onto it, a hole “jailbroken” with argon plasma coagulation (APC) at the ampulla to then allow biliary metal stent placement. For type 3 strictures, the least difficult type, biliary and duodenal stents can be placed, with the order being inconsequential. Complications of self-expanding metal stents may include bleeding, cholangitis, stent dysfunction, and perforation. Antireflux self-expanding metal stents can be used to prevent duodenal fluid reflux cholangitis [6]. 

While ERCP is the current standard minimally invasive treatment option for bilioduodenal strictures [7], an alternate management is EUS-guided lumen-apposing metal stents, which include EUS-gastrojejunostomy paired with EUS-choledochoduodenostomy or EUS-hepaticoduodenostomy. These EUS techniques have similar success rates as self-expanding metal stents and have the advantage of stent placement away from the sites of obstruction [7,8]. Despite advances over the past decade, these combined procedures, particularly the bilioenteric bypass portion, require subspecialized advanced endoscopists and can have high rates of post-procedure complications (7.7%-42.9%) including stent migration, reflux cholangitis, and biliary dysfunction (16.7%-71.4%) [7-9]. Combined biliary and duodenal self-expanding metal stenting has lower post-procedure complication rates which include post-sphincterotomy bleeding and mild pancreatitis (9.3%-12.5%) [2]. Type 1 strictures are particularly difficult to access for EUS-choledochoduodenostomy due to the location of the bile duct relative to the first portion of the duodenum [7]. A combination of stricture dilation, self-expanding metal stenting, lumen-apposing metal stenting, or percutaneous transhepatic wire and stent guidance can also be utilized to relieve concomitant biliary and gastric outlet obstructions depending on patient and anatomy circumstances.

## Conclusions

This case report shows successful acute palliative management of a patient with unresectable pancreatic adenocarcinoma who presented with dual gastric outlet and biliary obstruction, with endoscopic dual gastroduodenal stent placement and subsequently through-the-stent ERCP biliary stent exchange with self-expanding metal stents. Endoscopic dual gastroduodenal and biliary stent placement is a lower morbidity solution with higher clinical success compared to traditional surgical gastrojejunostomy and hepaticojejunostomy for palliative patients with concurrent malignant biliary and gastric outlet obstruction. ERCP is the current standard minimally invasive treatment of biliary obstruction in concomitant biliary and gastric outlet obstruction; however, EUS modalities are becoming more commonplace. Endoscopic treatment strategy of bilioduodenal strictures facilitates expedited return to quality of life and administration of systemic therapy.
